# The Role of Platelets in the Pathogenesis of Viral Hemorrhagic Fevers

**DOI:** 10.1371/journal.pntd.0002858

**Published:** 2014-06-12

**Authors:** Juan C. Zapata, Dermot Cox, Maria S. Salvato

**Affiliations:** 1 Institute of Human Virology, University of Maryland School of Medicine, Baltimore, Maryland, United States of America; 2 Molecular and Cellular Therapeutics School of Pharmacy, Royal College of Surgeons in Ireland, Dublin, Ireland; Centers for Disease Control and Prevention, United States of America

## Abstract

Viral hemorrhagic fevers (VHF) are acute zoonotic diseases that, early on, seem to cause platelet destruction or dysfunction. Here we present the four major ways viruses affect platelet development and function and new evidence of molecular factors that are preferentially induced by the more pathogenic members of the families *Flaviviridae*, *Bunyaviridae*, *Arenaviridae*, and *Filoviridae*. A systematic search was performed through the main medical electronic databases using as parameters all current findings concerning platelets in VHF. Additionally, the review contains information from conference proceedings.

## Introduction

Viral hemorrhagic fevers (VHFs) are a group of zoonotic diseases characterized by fever and bleeding disorders that can progress to shock and death. They are caused by different groups of viruses from the families *Flaviviridae*, *Bunyaviridae*, *Arenaviridae*, and *Filoviridae* [1], [2]. In addition to the VHF syndrome, these viruses have other common characteristics: they are enveloped viruses with ssRNA genomes and cytoplasmic replication; some of them share common viral sequences; and they use rodents, bats, or insects as natural reservoirs or vectors. Their circulation is geographically restricted by the habitats of their natural hosts, and human beings are incidental hosts. Outbreaks of VHF occur sporadically; consequently, these viruses are continuously emerging or re-emerging in places where they find the ideal conditions. Most of these viruses are pantropic, and dendritic cells (DC), monocytes and/or macrophages are among the targets. Some subsets of those cells are highly susceptible to VHF, producing a large amount of virus early after infection that modifies the cell's antigen-presenting and cytokine-producing functions [3]–[8]. This could contribute to the pathogenesis observed in most of the VHFs, in which severe cases are associated with uncontrolled viral replication and high levels of viremia. Unfortunately, more disease treatment possibilities and immune prevention options are needed (Table 1) [2].

**Table 1:** Common characteristics of hemorrhagic fever viruses.

Enveloped viruses with a ssRNA genome
Filovirus, Bunyavirus, and Arenavirus share some genomic sequences
Cytoplasmic replication
They are pantropic viruses that target primary dendritic and monocyte/macrophage cells
Sporadic outbreaks
Infection in human beings is generally asymptomatic or with flu-like symptoms
Severe cases are associated with high levels of virus in blood
Gastrointestinal and neurological symptoms
Rodents or insects are natural reservoirs or vectors
Continuously emerging or re-emerging
Geographically restricted by the presence of its natural host
Limited treatment options

## Impact and Epidemiology of VHF

**Figure 1:**
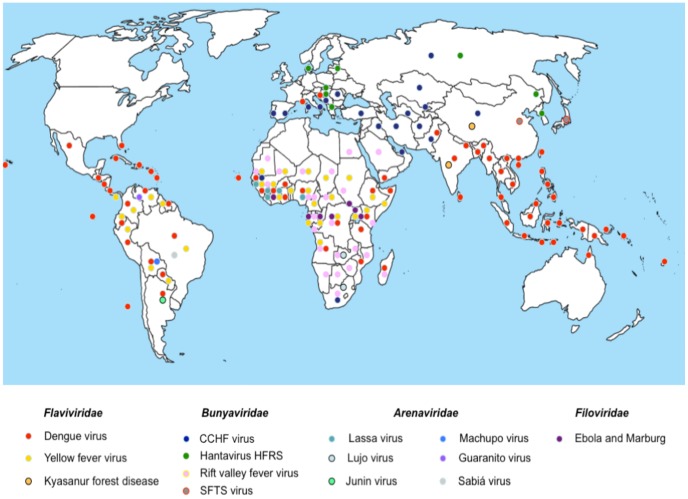
Geographical distribution of Viral Hemorrhagic Fevers (VHF). This map shows the global distribution of some members of the viral families related to hemorrhagic fever disease. CCHF stands for Crimean Congo Hemorrhagic Fever and SFTS for severe fever with thrombocytopenia syndrome.

The exploitation of new ecological niches by human beings and increased travel and trade have promoted the emergence or re-emergence of VHF around the world (Figure 1). All VHFs are considered zoonotic diseases and their transmission mechanism varies from virus to virus. For instance, infection may be through insect bites or through contact with meat or excreta from infected animals.

Prioritizing the impact of each of these VHF diseases is important in order to develop effective control and prevention methods and reduce the annual loss of life. Together VHF diseases affect more than 100 million people around the world and kill more than 60,000 annually. The epidemiology and transmission of each disease differs greatly (Table 2).

**Table 2:** Impact of hemorrhagic fever viruses.

Virus	Disease name	Cases/year	Estimated % fatality rate	Death/year
Dengue	Dengue Fever	50–100 million [131]		
	Dengue HF (DHF)	500,000 DHF [13]	1%–5%	22,000 DHF/DSS
	Dengue shock syndrome (DSS)			
Yellow Fever	Yellow Fever	200,000 [15]	15%–30%	30,000
Kyasanur forest disease virus	Kyasanur forest disease	100–500 [132]	2%–10%	1
Alkhumra virus	HF	11 [17], [18]	2%–10%	3
CCHF	Crimean-Congo HF	68 [31], [133]	30%–60%	3
Hantaan virus	Hemorrhagic fever with renal syndrome (HFRS)	200,000 [27]	1%–15%	10,000
Rift valley fever		34 [134]	1%	17
SFTS	Severe fever with thrombocytopenia syndrome (SFTS)	171 [34]	12%–30%	21
Lassa	Lassa fever	300,000 [19]	1%–15%	5,000
Lujo	HF	5	80%	4
Junin	Argentinian HF	300–800 [23]	15%–30%	300 (before vaccine)
Machupo	Bolivian HF	13 [24]	18%–20%	7
Guanarito	Venezuelan HF	85 [26]	23.1%	
Ebola	Ebola HF	56 [135]	50%–90%	37
Marburg	Marburg HF	18 [39]	25%	9
**Total**	Hemorrhagic fever = HF	**∼51**–**101 million**		**∼67,000**

Dengue virus (DENV) from the *Flaviviridae* is the most prevalent arthropod-borne VHF. Dengue is present on all continents, with new cases occurring and spreading to non-endemic areas in the United States and Europe (Figure 1; Table 2) [9]–[12]. Between 50–100 million cases of dengue fever and around 500,000 cases of dengue hemorrhagic fever (DHF) or shock syndrome (DSS) are reported every year, with estimated case fatality rates ranging between 1% and 5% [13]. Yellow fever (YF) virus, another flavivirus, is the second most globally distributed arthropod-borne disease, causing around 200,000 cases per year, with case fatality varying between 15% and 30%. Although there is an effective vaccine against YF disease, the number of infected people has increased in recent years, especially in urban Africa [14], [15]. Kyasanur Forest disease virus (KFDV), also a flavivirus, circulates in mammals and birds from India, Saudi Arabia, and Republic of China. KFDV is transmitted by forest ticks to humans and nonhuman primates, causing a severe febrile illness, sometimes with hemorrhagic symptoms [16]. Alkhumra virus, another flavivirus and a member of the mammalian tick-borne encephalitis group, is associated with acute VHF in Saudi Arabia, with fatality rates between 2% and 10% [17], [18].

Lassa fever virus (LASV) and Lujo virus (LUJV), from the Old World subset of the *Arenaviridae*, are both from Africa. LASV is transmitted by rodents and causes around 300,000 cases of Lassa fever (LF) each year in West Africa, with an overall fatality rate around 1% that can range between 15% and 30% during hospital outbreaks [19], [20]. LUJV was isolated in South Africa during a human outbreak characterized by nosocomial transmission and high fatality rates [21]. Since this outbreak, no new cases have been reported and its reservoir is still unknown. Junin (JUNV), Machupo (MACV), and Guanarito (GTOV) belong to the New World subset of the *Arenaviridae* and, together, are known as South American hemorrhagic fever (HF) viruses. These viruses circulate in rodents and occasionally are transmitted to human beings through the urine or feces of their carriers. JUNV causes Argentinian HF (AHF), with case fatality between 15% and 30%. Since the introduction of the attenuated Candid #1 vaccine the number of AHF cases has decreased dramatically [22], [23]. MACV is responsible for Bolivian HF (BHF), with 13 cases of the disease reported in 2012 and seven deaths [24]. GTOV causes Venezuelan HF in the western part of the country, with an estimated case-fatality rate of 23% [25]. Between 2011 and 2012, more than 85 people acquired this disease [26].

Hantaan virus, Seol virus (SEOV), Crimean-Congo Hemorrhagic Fever (CCHF) virus, Rift Valley Fever virus, and Severe fever with Thrombocytopenia Syndrome (SFTS) virus belong to the family *Bunyaviridae*. These viruses gained importance in the last decade because they have been reported in places in which they are not endemic, with a tendency to continue expanding their circulation to other regions. Hantaan virus and SEOV, harbored and transmitted by contact with infected rodent excreta, cause close to 200,000 Hemorrhagic Fever with Renal Syndrome (HFRS) cases each year, with case fatality rates from 1% to 16% in Asia and the Far East Region of the Russia Federation [27]. *Nephropathia epidemica*, a mild form of HFRS caused by Puumala virus, is the most prevalent hantaviral disease in Western and Central Europe. All these viruses are widely distributed and continue to expand to new regions [28], [29]. CCHF is a sporadic but often-fatal, tick-borne disease that is expanding through Europe, Asia, and Africa [30], [31]. Rift Valley Fever virus is transmitted by mosquitos and also causes sporadic outbreaks of hemorrhagic fever in some African countries and, more recently, the Arabian peninsula, with variable mortality rates [32], [33]. SFTS virus has recently emerged in China and Japan with mortality rates between 12% and 30% [34], [35]. SFTS life cycle is still unknown; however, seroconversion and virus isolation involve domesticated animals as reservoirs and ticks as vectors [36]. It has been suggested that SFTS can also be transmitted through blood or personal contact with an infected patient [37].

Ebola and Marburg viruses (from the family *Filoviridae*) cause sporadic outbreaks amongst human and nonhuman primates in Central Africa (and recently, West Africa), with very high case fatality rates, ranging from 25% to 90% [38], [39]. These viruses are most often transmitted by direct contact with infected animals or people. The natural reservoirs for filoviruses are thought to be bats [38], [40].

Only two vaccines are licensed to prevent VHF: YF17D and Candid#1, live-attenuated versions of the YF virus and JUNV. Unfortunately, the VHF-endemic areas are expanding, with the aggravation that treatments are limited or nonexistent. For this reason, it is important to continue research and development for new prevention and control options.

## Clinical Signs and Laboratory Findings

VHF signs and symptoms range from asymptomatic infection to life-threatening disease. Initially, patients develop a nonspecific febrile syndrome (flu-like) that can include chills, malaise, headache, backache, arthralgia, myalgia, retro-orbital pain, anorexia, nausea, vomiting, diarrhea, cough, and sore throat [41]–[46]. At this stage the clinical signs resemble other infectious diseases, making early VHF clinical diagnoses impossible. Some cases progress to severe disease characterized by systemic vascular damage that, depending on the virus, will manifest as subcutaneous bleeding (flushing, conjunctivitis, peri-orbital edema, petechiae, or ecchymosis), positive tourniquet test, hypotension and internal organ bleeding, hematemesis, and melena [41]–[46]. The cause of bleeding in most VHF diseases is a disseminated intravascular coagulation (DIC) that depletes the coagulation factors, inducing massive plasma leakage, hypovolemia, and shock. A prolonged shock state leads to multiple organ failures and, in some cases, death. However, the loss of blood is rarely the cause of death (with the exception of filoviruses) [41]–[46].

The hallmark characteristic of all VHF is a decrease in platelet numbers and/or function. This decrease is usually accompanied by an increase in fibrinogen degradation products, prothrombin time (PT), and partial thromboplastin time (PTT). At least five days after the onset of fever, there is also a marked leucopenia and high viral loads that are associated with fatal outcomes. Another common finding is an increase in the alanine aminotransferase (ALT) and aspartate aminotransferase (AST) enzymes, primarily due to liver damage [41]–[46].

## VHF Pathogenesis

Based on some clinical and laboratory findings (Table 3), different pathogenic mechanisms have been proposed for each VHF, including depletion of hepatic coagulation factors, cytokine storm, increased permeability by vascular endothelial growth factor, complement activation, and DIC. In spite of the differences seen with each VHF, there is a large body of evidence indicating that viral replication and host immune responses play an important role in determining disease severity and clinical outcome [47]–[50]. HF viruses can establish nonlytic replication, or “virus factories,” in monocytes and/or macrophage and DC [47], [49]. Viral replication subverts the function of these cells, as well as the function of uninfected bystander cells, to undermine the innate immune response, such as interferon (IFN) production. This leads to uncontrolled viral replication and to a lack of specific antiviral responses in the host [47]–[50]. These cells might also act as vehicles to carry the virus to its replication sites, such as endothelium, liver, spleen, and other organs, leading to the pantropism of most hemorrhagic fever viruses [47]–[50]. Whereas the early replication stage is characterized by a lack of IFN production and IFN-responsive gene expression, later viremic stages reveal an unregulated release of pro-inflammatory cytokines, such as tumor necrosis factor alpha (TNF-α), IL-10, IL-1Rα, and soluble TNF-R, that could contribute to immunosuppression and increased vascular permeability, leading to hemorrhagic signs [51], [52].

**Table 3:** Hemorrhagic fever virus findings related to coagulation disorders.

Viral Family/Genus	Leukopenia and immune-suppression	Thrombo-cytopenia	Platelet altered function	Reduced levels of coagulation factors	DIC	Endothelial dysfunction	Hepatocyte dysfunction
*Flaviviridae*							
Dengue	+	+	+	+	+	+	+
Yellow fever	+	+	?	+	+	+	+
Kyasanur forest	+	+	?	+	+	?	+
*Bunyaviridae*							
CCHF	+	+	?	+	+	+	+
Hantavirus HFRS	I	+	+	+	+	+	+
Rift valley fever	+	+	?	+	+	+	+
SFTS	+	+	?	+	+	−	+
*Arenaviridae*							
Lassa	+	+/N	+	−	−	+	−
Lujo	+	+			+gp		
Junín	+	+	+	−	+/−	+	−
Machupo	+	+	+	+	+	+	+
Guaranito	+	+	+	+	+	+	+
Sabia	+	+					
*Filoviridae*							
Ebola	+	+	+	+	+	+	+
Marburg	+	+	+	+	+	+	+

N = Normal, I = increased, gp = guinea pigs. This table is modified from [136].

Thrombocytopenia (reduction of platelet numbers) and depressed immune responses are hallmarks of VHF, and platelets are involved in both processes; therefore, this review will focus on the platelet's role in VHF pathogenesis.

## Platelet Structure and Functions

**Figure 2:**
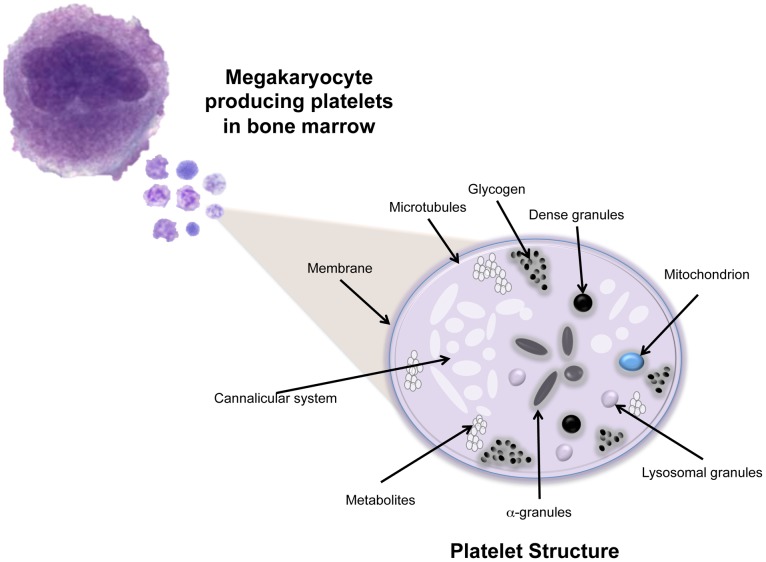
Platelet structure. Platelets have multiple surface receptors, a cannalicular system, microtubules, mitochondria, three types of granules (lysosomal, alpha, and dense), and deposits of small factors like glycogen (Figure 2 and 3) [54]. Whereas dense granules contain factors that potentiate platelet activation, α-granules contain growth factors and clotting proteins that contribute to hemostasis [130].

Platelets, also known as thrombocytes, are small, 2–3 µm, enucleated cell fragments derived from the cytoplasm of large, 100 µm, megakaryocytes (MKs) located in the bone marrow (Figure 2) [53]. Each MK sheds thousands of platelets into the blood stream, making them the second-most-abundant cell type in peripheral blood. Platelets' most-known function is to maintain the integrity of the vascular system [53]; however, their function as immune elements is becoming more evident.

**Figure 3:**
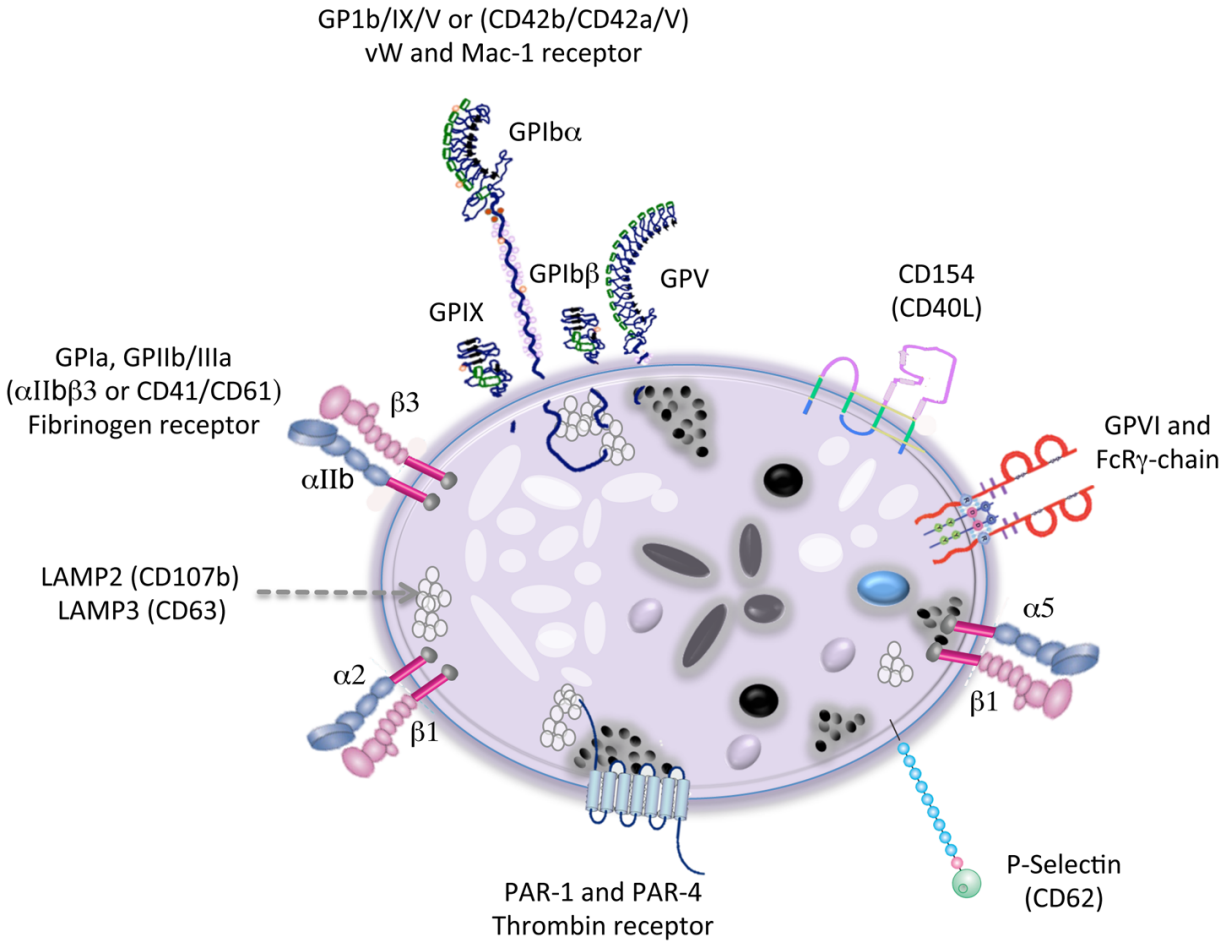
Platelet content. Membrane glycoproteins of platelets include GPIa, GPIIb/IIIa (aIIbb3), or VLA-5 (fibrinogen receptor); GPIb/IX/V (vW and Mac-1 receptor); GPIc'-IIa or VLA-6 (laminin receptor); and a2b1 GPVI (Collagen receptor). **Alpha granules** contain P-selectin; platelet factor 4; transforming factor-b1; chemokines; proteoglycan; platelet-derived growth factor; a2-plasmin inhibitor; vitronectin; laminin; CD63; TGFbeta; CLEC-2; thrombospondin; fibronectin; B-thromboglobulin; vWF; fibrinogen; coagulation factors V, XI, and XIII; integrins; thrombocidins; proteases; thrombin; prothrombin; kininogens; immunoglobulin family receptors; leucine-rich repeat family receptors; and other proinflammatory and immune-modulating factors. **Dense granules** hold ADP, ATP, calcium, serotonin, histamine, dopamine, phosphate, eicosanoids. **Receptors for primary agonist include** P2X, P2Y_1_, and P2Y_12_ (ADP); TPa-R and TPb-R (TXA_2_); PAR-1 and PAR-4 (thrombin); PAFR (platelet-activating factor); 5-HT_2A_ (Serotonine); epinephrine receptors (catecholamines); Fc and complement C3_a_/C5_a_ receptors; and TLRs, CD40, CD40L, ICAM-2, DC-SIGN, JAM-A, and FcγRII. Between the platelet **Metabolites** are TXA_2_, sphingosine-1-phopate, PAF, glycogen, platelet factor 4 (PF-4), RANTES, connective tissue activating peptide 3 (CTAP-3), platelet basic protein, thymosin β-4 (Tβ-4), fibrinopeptide B (FP-B), and fibrinopeptide A (FP-A).

Under specific stimulus, such as tissue damage, platelets are activated by extracellular matrix proteins, such as collagen and von Willebrand factor. Their irregular shapes swell to a compact spherical form with projections containing glycoprotein receptors that help them to attach to each other and to cells at wound sites [54]. After activation, platelets release granule contents and coagulation factors that further enhance their activation and cell attachment (Figure 3). This process results in the formation of an effective plug at the site of injury that supports formation of a fibrin network and stops bleeding [55].

There are some pathological processes in which the normal number of circulating platelets can decrease (thrombocytopenia), increase (thrombocytosis), or diminish in function (thrombasthenia) with or without hemostatic manifestations. The study of those conditions showed that in addition to their role in homeostasis, platelets are important mediators of inflammation and innate immunity [56]. Sub-products generated from microorganisms, from complement fixation reactions, or from expression of receptors such as Fc and C3_a_/C5_a_ can attract platelets to the site of infection or injury where they are activated by thrombin and bind to vascular endothelial cells and leukocytes. Platelets can also bind pathogens directly, recognizing them through Toll-like receptors (TLRs), and can alert other cells via the innate immune response [57]. Platelets can directly release antimicrobial factors such as platelet factor 4 (PF-4), RANTES, connective tissue activating peptide 3 (CTAP-3), platelet basic protein, thymosin β-4 (Tβ-4), fibrinopeptide B (FP-B), fibrinopeptide A (FP-A), superoxide, hydrogen peroxide, and hydroxyl free radicals. Additionally, platelets participate in antibody-dependent cell cytotoxicity against microbial pathogens [58].

In conclusion, any pathogenic state affecting platelets will not only impact hemostasis but also will modify the immune response to infection. Although there are some advances in understanding the specific platelet activation mechanisms induced by some microorganisms, little is known about these interactions and their role in the pathogenesis of VHF.

## Direct and Indirect Virus–Platelet Interactions

Several microorganisms or molecules derived from them interact with platelets and affect their function [55], [59], [60]. Virus–platelet interactions were initially described around 50 years ago with some in vitro experiments using influenza virus and other myxoviruses [61], [62]. Since then, other viruses have been reported to interact not only with platelets but also with MKs [63]–[68]. From these interactions, platelet numbers or functions can be compromised, and little is known about the exact mechanisms. In the case of VHF, recent publications described pathogenic mechanisms involving the role of platelets in homeostasis as well as their role in the initial immune responses in animals and human beings. The next paragraphs will describe different ways in which hemorrhagic fever viruses affect platelets.

There are four major causes of thrombocytopenia or platelet malfunction induced by viruses. These include direct effects of viruses on platelets, immunological platelet destruction, impaired megakaryopoiesis, and MK destruction (Table 4) [68]. In the VHF diseases, the most common mechanism of thrombocytopenia is platelet disappearance from damaged tissues or generalized virus-induced DIC, in which coagulation factors are depleted. This common pattern does not seem to apply to LASV. Additionally, there is some evidence that viruses not belonging to the hemorrhagic fever group (such as varicella, herpes zoster, smallpox, rubella, measles, cytomegalovirus, rotavirus, adenovirus, and HIV), under specific circumstances, can also cause hemostatic problems and can be associated with DIC [66], [69]–[72]. More work is necessary to clarify the role of the platelet–virus interaction in coagulopathies and in DIC induced by both hemorrhagic and nonhemorrhagic viruses.

**Table 4:** Four main mechanisms by which HFVs induce thrombocytopenia.

Virus	aDestruction of platelets by direct interaction	bImmunological destruction of platelet-viruses complexes	cMegakaryocyte impairment	dInhibition of platelet function
Dengue	Not yet described	Platelet-virus associated IgM or IgG	Destruction of early blast and hematopoietic DENV-infected cells by macrophages and DC	Platelet IgM auto-antibodies inhibit ADP-induced platelet aggregation
		Platelet IgM auto-antibodies binding the C3-complement molecule		
Hantavirus HFRS	α_v_β_3_ and α_IIb_β_3_	Not yet described	Destruction of infected MK by CTL [114]	Defective platelet aggregation [96]
*SFTS* virus	Not yet described	Increased platelet-virus phagocytosis [97]	Not yet described	Not yet described
Lassa virus	Not yet described	not yet described	Not yet described	Unknown platelet aggregation inhibitor [127], [137]
Junín virus	Not yet described	Not yet described	Increased bone marrow type I IFN levels	Unknown platelet aggregation inhibitor [128]
Ebola	Not yet described	Not yet described	Not yet described	Elevated levels of type I IFN [138]
				Defective aggregation of surviving platelets [139]

a Destruction of platelets by direct interaction: HFV can bind platelets directly causing activation and granule release.

b Immunological destruction of platelet-virus complexes: Thrombocytopenia can be mediated by macrophages sequestration of platelet-virus complexes at the infection site or/and in the spleen, platelets-virus-leukocyte aggregation and subsequent phagocytosis by macrophages or destruction mediated by platelet-virus associated antibodies.

c Megakaryocytes or Megakaryocyte precursors impairment: HFV can infect megakaryocytes or their precursor causing reduction in platelets number or impairment in their function.

d Inhibition of platelet function: Some unidentified soluble factors present in plasma from infected patients can inhibit aggregation of platelets from healthy individuals.

## Platelet–Virus Binding

Platelets bind viruses through different receptors, such as β-3 integrins or TLRs, and platelets are known to express TLR2, TLR4, and TLR9 [73], [74]. In severe sepsis, there are coagulation abnormalities and DIC that are thought to be due to TLR signaling in platelets [57]. Bacterial stimulation of platelet TLR2 increased P-selectin surface expression, activation of integrin *α*_IIb_ *β*_3_, generation of reactive oxygen species, and formation of platelet–neutrophil heterotypic aggregates in human whole blood [75]. Members of the family *Bunyaviridae* have been shown to bind platelets; specifically, hantaviruses bind to α_v_β_3_ or α_IIb_β_3_ integrins expressed on platelets and endothelial cells, contributing to viral dissemination, platelet activation, and induction of endothelial cell functions. These events reduce the number of circulating platelets and increase vascular permeability [74], [76].

Platelets can also be activated by exposure to virus-infected cells. When HUVEC cells are infected with DENV, and then exposed to human platelets, the platelets become activated and bind to the endothelial cells [77]. Several viruses have shown in vitro affinity for the integrin receptors on both platelets and vascular endothelial cells: e.g., α_V_β_3_ binds coxsackievirus A9, human adenovirus type 2, foot-and-mouth disease virus, echovirus 9, and human paraechovirus 1 [78]–[82]; α_V_β_3_ and α_IIb_β3 bind hantavirus [76]; α_2_β_1_ interacts with human echovirus 1 and rotavirus [83], [84]. LASV and most isolates of the Old World arenaviruses use α-dystroglycan (α-DG) [85], [86]. By using this receptor, those viruses can infect endothelial cells without cytotoxicity. However, as a result of interactions with virus particles or virus-infected cells, platelets will be activated to adhere to endothelial cells, thereby reducing the number of circulating platelets, altering endothelial cell function, and increasing vascular permeability.

There are other viral receptors that are present in cells of the immune system, such as Clec-2 and DC-SIGN that binds HIV [68], [87]; DC-SIGN also shows affinity for other lentiviruses and DENV [88], [89]. DC-SIGN, Axl, and Tyro3 are Ebola virus and LASV receptors [90]–[93]. Ebola virus requires the cholesterol transporter Niemann-Pick C1 (NPC1) for cell entry [94]. This receptor is found in cells that are affected by HFV, such as gastrointestinal epithelial cells, and hepatocytes. Although all these receptors that bind HFV in target cells are also present in platelets and MKs (and, in some cases, have been shown to mediate virus internalization), it remains unknown whether they interact with platelets in vivo or if virus internalization leads to successful replication or even propagation of these viruses.

After binding viruses, platelets can engulf them and process them in small endocytic vesicles that are later fused with secretory granules that destroy the virus [61], [67], [95]. However, virus destruction is not always successful, and infected platelets can contribute to the dissemination of an infection [68] and to platelet dysfunction, as seen in HFRS [96].

## Immunological Destruction of Platelets or Their Precursor Cells

**Figure 4:**
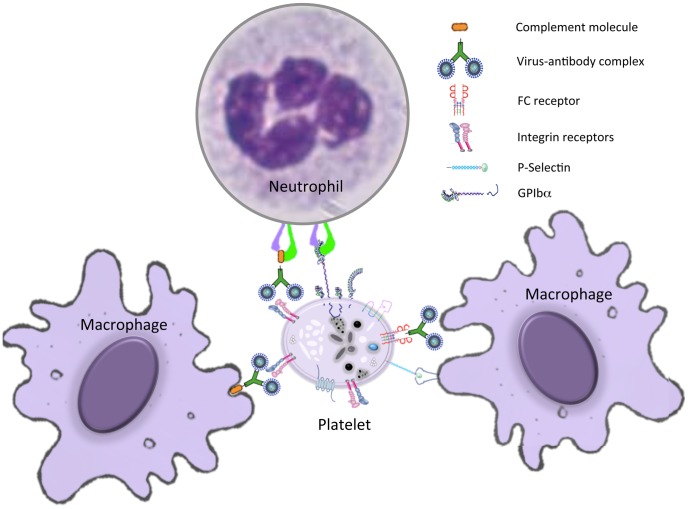
Immunological destruction of platelets. Platelets can interact with macrophages and neutrophils at the infection site and/or in the spleen through immuno-complexes or directly by cellular-ligand interactions. These interactions lead to either platelet sequestration or platelet destruction mediated by the immune system.

There are at least three different ways in which the immune system can contribute to the reduction of platelet numbers (Figure 4). The first one is platelet sequestration by macrophages at the infection site and/or in the spleen. Viral activation of platelets induces an increased expression of P-selectin that functions as a receptor for macrophages [68]. As seen in SFTSV, another member of the *Bunyaviridae* associated with coagulation disorders, binding to platelets induces macrophage phagocytosis of platelets in the spleen, causing a decrease in circulating platelet numbers [97].

A second mechanism of platelet depletion is platelet-leukocyte aggregation and subsequent phagocytosis by macrophages. Activated platelets recruit leukocytes to inflamed endothelium, and neutrophils are the most frequent and rapidly recruited cells to the site of injury or inflammation [98], [99]. Platelet-leukocyte interaction is mediated by the platelet GPIba protein and the leukocyte macrophage-1 antigen protein (MAC-1), also known as α_M_β_2_ integrin, complement receptor 3 (CR3) protein, CD11B, and Integrin alpha-M (ITGAM). The mRNA for this integrin is 4-fold up-regulated in peripheral blood mononuclear cells (PBMC) exposed to LASV in comparison to PBMC exposed to a benign virus, ML29 [100]. ML29 is a nonpathogenic reassortant between Lassa and Mopeia viruses, with the L genome segment from Mopeia and the S segment from LASV. It is tempting to speculate that the up-regulation of this integrin on leukocytes promotes their aggregation with platelets and has a role in LASV pathogenesis, but many more studies are needed.

Platelets secrete chemokine proteins, such as CXCL1, CXCL4 (PF4), CXCL4L, CCL5, CXCL7, CXCL8 (IL-8), CXCL12, CXCR2, CCL2, CCL3, and CCL5, that promote angiogenesis and chemotaxis [101]. CXCL4 and CCL5 specifically induce monocyte migration and binding to the inflamed endothelium [102]. In a similar way, crosslinking of P-selectin glycoprotein ligand 1 (PSGL-1) on monocytes by P-selectin expressed in platelets and endothelial cells enhances monocyte recruitment to the activated endothelium [101] as seen in adenovirus or DENV-endothelial infected cells [77], [103]. This cascade of interactions not only regulates leukocyte trafficking to the inflamed site but also increases local platelet sequestration, phagocytosis and lysis.

A third mechanism of platelet depletion is their destruction mediated by platelet-virus associated antibodies. In a secondary DENV infection, antibodies against the prM viral protein facilitate efficient binding of immature particles to Fc-receptor-expressing cells [104]. Platelets express FcγRIIa, which binds these anti-prM-DENV complexes and renders them suceptible to destruction by the immune system. In addition, platelet-DENV complexes also bind the C3-complement molecule and platelet-associated IgM or IgG antibodies, targeting them for clearance by cells of the immune system [105], [106]. Furthermore, DENV antibodies reacting against platelet glycoproteins can mediate destruction of platelets by complement or by the monocyte-macrophage system. Such antibodies could also suppress MK proliferation and maturation. Specifically, IgM anti-platelet autoantibodies not only induce platelet lysis via complement activation but also inhibit ADP-induced platelet aggregation [107].

## Suppression of Megakaryocyte and Platelet Development

Several viruses, like HIV-1, HCV, and human CMV, are able to replicate in MKs, and those infections are associated with thrombocytopenia and other thrombotic disorders [108]–[110]. Since platelets are derived from MKs, they express similar membrane receptors, therefore platelets may bind and internalize similar viruses. However, there are also differences in receptor expression and metabolic pathways among MK-precursors, mature MKs, and platelets that make them susceptible or resistant to specific viral infections. For instance, immature MKs express CD4 antigen on their surface, making them susceptible to HIV infection, while mature MKs and platelets do not express CD4 [68]. HHV-7 has the capacity to infect CD61+ megakaryoblast cells, increasing apoptosis of these cells and causing an impaired megakaryopoiesis [111]. Furthermore, HHV-6 is able to infect CD34+ hematopoietic progenitors cells, thereby suppressing thrombopoiesis [112]. DENV decreases platelet numbers by inhibiting progenitor cell development in the bone marrow. In vitro, early blast and hematopoietic cells in an intermediate state of differentiation were abortively infected, killed, and eliminated by DC phagocytosis [113]. Pathogenic hantaviruses can infect MKs, in contrast to non-pathogenic hantaviruses. HTNV-infected MKs show no increased apoptosis or problems with differentiation, but they do show increased surface expression of HLA, making them good CTL targets and resulting in acute thrombocytopenia [114]. It has also been proposed that LCMV-induced thrombocytopenia in mouse is associated with the effect of IFN-α/β on MKs, leading to the generation of altered platelets [115].

Another mechanism that affects platelet formation is the decreased production of thrombopoietin (TPO) due to liver damage in HCV infection that results in suppression of development in the bone marrow [116].

## Inhibition of Platelet Function

The integrin β_3_ (ITGB3 or CD61+) subunit in platelets can combine with different partners, resulting in different integrin receptors with multiple functions. The integrin α_v_β_3_ is a receptor for cytotactin, fibronectin, laminin, matrix metalloproteinase-2, osteopontin, osteomodulin, prothrombin, thrombospondin, vitronectin, and von Willebrand factor. Integrin α_IIb_β_3_ is a receptor for fibronectin, fibrinogen, plasminogen, prothrombin, thrombospondin, and vitronectin. It mediates platelet–platelet interaction by binding of soluble fibrinogen. In acute DENV infection there is an increase in CD61+ on platelets and MKs containing viral proteins and RNA that suggests a link with platelet dysfunction and low platelet counts [105].

Arenaviruses have several mechanisms for inhibiting platelet function. In the LCMV-infected mouse model for thrombocytopenia, ITGB3^−/−^ mice developed a severe hemorrhagic anemia after infection, even with normal platelet counts, suggesting a key role of ITGB3 in protecting against bleeding [115]. In vitro exposure of human PBMC to a pathogenic arenavirus (LASV) increases the expression of ITGB3 mRNA by 12-fold in contrast to ML29, the attenuated derivative of LASV mentioned earlier, that does not affect ITGB3 mRNA expression [100]. How LASV is affecting the function of ITGB3 in vivo remains to be explored, and the protection seen in the mouse model, combined with the increased expression in human PBMC exposed to a pathogenic virus, suggests that ITGB3 is playing an important role in arenavirus infections.

It is noteworthy that there are important differences between the LCMV mouse model for thrombocytopenia, in which IFN-α/β mediates the decrease in platelet numbers, platelet dysfunction, and bleeding, and the LCMV primate model, in which the IFN-α/β expression is not remarkable and yet there are bleeding signs [117]–[119]. Similarly, LCMV infection in humans more closely resembles a JUNV infection, with marked thrombocytopenia and platelet dysfunction [115], than a LASV infection, which is characterized by variable changes in platelet numbers and constant compromise of platelet function. IFN-α/β is affecting platelets either by a direct effect on MKs, leading to the production of altered platelets, or by the up-regulation of endothelial cell-derived platelet inhibitors, such as nitric oxide and prostacyclin, that indirectly contribute to the observed platelet dysfunction [115]. In vitro infections of MKs with JUNV showed impaired thrombopoiesis by decreasing pro-platelet formation, platelet release, and P-selectin externalization via a bystander effect. All these effects were associated with increased levels of type I IFN [120]. In addition, endothelial cells infected with a pathogenic JUNV strain showed an increase in nitric oxide (NO), endothelial nitric oxide synthase (eNOS), and PGI2 production [121]. Similarly, Pichinde virus (PICV, another arenavirus) induces endothelial cell permeability through the production of NO [122]. It is expected that the up-regulation of NO by JUNV will have similar effects on the permeability of endothelial cell layers.

It has been demonstrated that thrombomodulin (THBD) binds thrombin and blocks the ability of thrombin to activate platelets. THBD also completely blocked tissue factor-induced microparticle formation [123]–[124]. Additionally, platelets contain functional THBD [125], and the platelet alpha-granule protein PF4 enhances activated protein C (APC) generation by soluble and membrane THBD in endothelial cells [126]. PBMC exposed to a pathogenic arenavirus (LASV) increased expression of THBD mRNA by 14-fold. In contrast, the attenuated ML29 inhibits THBD mRNA expression. This pattern was observed also at the protein level in PBMC and DC for both soluble and membrane-bound THBD [100]. Serum from LASV-infected patients suffering hemorrhagic symptoms contains an unidentified soluble factor able to inhibit aggregation of platelets from healthy individuals [127]. Similarly, there is evidence of an inhibitor in plasma of JUNV-infected patients [128] that is not neutralized by plasma containing high titers of protective antibodies against JUNV [129], suggesting that this is not a virus-derived molecule. This factor could be THBD that, after an overexpression induced by LASV, inhibits platelet activation through thrombin binding and, at the same time, prevents thrombin production by APC. As a consequence, there is an increased susceptibility to hemorrhagic episodes during LF disease [100].

In conclusion, HFVs all induce homeostatic abnormalities, inhibit the antiviral immune response, and display high levels of viremia. Platelets are peripheral blood elements that play roles in both homeostasis and inflammation. Platelet–HFV interactions are poorly understood and seem to be different in each disease, but there are similar mechanisms underlying all VHF pathogenesis. The identification of such mechanisms can lead to the development of new treatment options for this group of diseases that kill millions of human beings each year.

**Key Learning Points**

- Viral hemorrhagic fevers (VHF) are a group of zoonotic diseases characterized by fever and bleeding disorders that can progress to shock and death.
- Thrombocytopenia (reduction of platelet numbers) and depressed immune responses are hallmarks of VHF; platelets are involved in both processes, and their function is also compromised in these types of infections.
- Platelet–HFV interactions are poorly understood and seem to be different in each disease, but some mechanisms are common to all VHF pathogenesis.
- It is important to understand the role of platelets in each viral hemorrhagic fever disease to understand the pathogenesis mechanisms and identify new possible ways to prevent or treat this group of diseases.

**Five Key Papers in the Field**

1. Yeaman MR (1997) The role of platelets in antimicrobial host defense. Clin Infect Dis 25: 951–968; quiz 969–970.
2. George JN (2000) Platelets. Lancet 355: 1531–1539.
3. Youssefian T, Drouin A, Masse JM, Guichard J, Cramer EM (2002) Host defense role of platelets: engulfment of HIV and Staphylococcus aureus occurs in a specific subcellular compartment and is enhanced by platelet activation. Blood 99: 4021–4029.
4. Patel SR, Richardson JL, Schulze H, Kahle E, Galjart N, et al. (2005) Differential roles of microtubule assembly and sliding in proplatelet formation by megakaryocytes. Blood 106: 4076–4085.
5. Loria GD, Romagnoli PA, Moseley NB, Rucavado A, Altman JD (2013) Platelets support a protective immune response to LCMV by preventing splenic necrosis. Blood 121: 940–950.
